# Haplotype Analysis and Gene Pyramiding for Pre-Harvest Sprouting Resistance in White-Grain Wheat

**DOI:** 10.3390/ijms26020728

**Published:** 2025-01-16

**Authors:** Haibin Dong, Cheng Kou, Lin Hu, Yan Li, Yuhui Fang, Chaojun Peng

**Affiliations:** Institute of Crop Molecular Breeding, Henan Academy of Agricultural Sciences/Key Laboratory of Wheat Biology and Genetic Breeding in Central Huanghuai Area, Ministry of Agriculture/Key Laboratory for Wheat Germplasm Resources and Genetic Improvement in Henan Province, Zhengzhou 450002, China; donghb1021@163.com (H.D.); liyanly7812@163.com (Y.L.); fang.yuhui@163.com (Y.F.); pengcj1023@163.com (C.P.)

**Keywords:** wheat, pre-harvest sprouting, molecular markers, haplotype combination

## Abstract

The Huanghuai winter wheat region, China’s primary wheat-producing area, predominantly cultivates white-grained wheat. Pre-harvest sprouting (PHS) significantly impacts yield and quality, making the breeding of PHS-resistant varieties crucial for ensuring China’s wheat production security. This study evaluated the PHS rate of 344 white-grained wheat varieties over two consecutive growing seasons (2022/2023 and 2023/2024). Furthermore, it analyzed the effects of allelic variations and their combinations in six genes (Tamyb10, TaDFR, TaMKK3-A, TaGASR34, Tasdr, and TaMFT) on PHS resistance. Results revealed average PHS rates of 66.1% and 64.4% for the two growing seasons, with coefficients of variation of 39.1% and 40.2%, respectively, and a narrow-sense heritability of 0.72. These findings indicate substantial genetic variation and relatively high genetic stability within the tested materials. Among the six molecular markers examined, the superior haplotype GS34-7Bb exhibited the lowest average PHS rate (41.9%) over two growing seasons, demonstrating the strongest PHS resistance. Analysis of different haplotype combinations identified two advantageous genotypes for PHS resistance in white-grained wheat: TaMKK3-Ab + GS34-7Bb + Tasdr-2Aa + TaMFT-A1b (average PHS rate: 20.8%) and TaMKK3-Ab + GS34-7Bb + Tasdr-2Ab + TaMFT-A1b (average PHS rate: 34.2%). Notably, the distribution frequency of superior haplotypes of PHS-related genes and these two advantageous haplotype combinations showed varying degrees of decline over time.

## 1. Introduction

Pre-harvest sprouting (PHS) is a global agricultural challenge, particularly severe in China’s middle and lower reaches of the Yangtze River winter wheat region, the southwestern winter wheat region, and the northeastern spring wheat region [1,2,3]. PHS in wheat has profound and far-reaching consequences, significantly degrading grain quality, altering biochemical composition and technological properties, adversely affecting grain storability, and compromising seed vigor for subsequent growing seasons. These cascading effects result in substantial economic losses throughout the wheat production and processing chain [4,5]. Red-grained wheat varieties generally exhibit superior PHS resistance compared to their white-grained counterparts [6]. The Huang-Huai wheat region, crucial for China’s food security, predominantly cultivates white-grained wheat varieties, which generally show poor PHS resistance. From late May to early June 2023, the western Huang-Huai region experienced prolonged, intense rainfall coinciding with wheat maturation and harvest, resulting in widespread fungal contamination and PHS. Consequently, in Henan province, one of the most severely affected areas, the per unit area yield and total production of summer grain decreased by 7.0% and 6.9%, respectively, compared to 2022 levels [7]. This event has been characterized as the most severe ‘rain-induced field damage’ weather in the province in over a decade [8]. These circumstances underscore the urgent need for breeding and deploying PHS-resistant white-grained wheat varieties.

Wheat PHS resistance is a complex quantitative trait influenced by multiple genetic and environmental factors. The polygenic nature of this trait necessitates sophisticated breeding strategies, among which marker-assisted selection (MAS) has emerged as a rapid and efficient approach. Recent advances in wheat genomics have facilitated the identification and characterization of numerous PHS-related genes, leading to the development of associated molecular markers [9]. Several key genes and their associated markers have been identified for PHS resistance. TaDFR and Tamyb10 regulate seed coat pigmentation and are candidate genes for the R locus, which is closely associated with seed coat-imposed dormancy [10]. Bi et al. [11] identified a single nucleotide polymorphism in the TaDFR-B homeolog and developed corresponding molecular markers. Himi et al. [10] cloned the Tamyb10-A1, Tamyb10-B1, and Tamyb10-D1 genes and developed associated markers, with Tamyb10-D1 exerting the most significant influence on PHS resistance [12]. The TaMKK3-A gene, involved in ABA signal transduction, plays a crucial role in modulating seed dormancy. Torada et al. [13] identified an A/C allelic variation of 665 bp upstream of the start codon and developed molecular markers for screening PHS-resistant germplasm. The TaMFT gene, part of the MFT gene family known to be pivotal in seed dormancy regulation [14], has also been implicated in PHS resistance. Jiang et al. [15] discovered a 33 bp insertion/deletion in the promoter region of TaMFT-3A and developed the TaMFT-A2 marker for PHS resistance screening. Furthermore, Cheng et al. [16] elucidated the role of TaGASR34 on chromosome 7B in seed dormancy. Through comparative sequencing of contrasting genotypes, they identified six SNPs in the promoter region and developed the GS34-7B CAPS marker for PHS resistance selection. These molecular genetic advances provide a robust foundation for enhancing PHS resistance in white-grained wheat varieties through precision breeding approaches.

In this study, we screened molecular markers associated with six PHS-related genes, Tamyb10, TaDFR, TaMKK3-A, TaGASR34, Tasdr, and TaMFT, selecting markers with the potential for effective high-throughput detection. Using a natural population of 344 white-grained wheat germplasm varieties and PHS phenotypic evaluations conducted over two growing seasons, we elucidated the distribution patterns of these genes within white-grained wheat. Furthermore, we analyzed the effects of different allelic variations and their combinations on PHS resistance. This comprehensive approach aims to provide essential genetic resources and theoretical foundations for breeding new PHS-resistant white-grained wheat varieties.

## 2. Results

### 2.1. Phenotypic Evaluation of Wheat Pre-Harvest Sprouting (PHS)

PHS resistance was phenotypically evaluated in 344 wheat varieties using the whole-spike sprouting method (Table S2). PHS rates exhibited substantial variation across both growing seasons, ranging from 2.7% to 100% in 2022/2023 and 4.4% to 100% in 2023/2024. The average PHS rates were 66.1% and 64.4% for the respective growing seasons, with coefficients of variation of 39.1% and 40.2%. The calculated narrow-sense heritability of 0.72 indicates considerable genetic variation among the test materials and relatively high genetic stability. Based on the two growing seasons’ average PHS rates, the varieties were classified into three categories: highly resistant (PHS rate < 20%), moderately resistant (21% ≤ PHS rate ≤ 40%), and susceptible (PHS rate > 40%). The distribution of varieties across these categories varied between the two growing seasons. In the 2022/2023 growing season, we identified 19 varieties (13 landraces, 6 cultivars) as highly resistant, 51 varieties (25 landraces, 26 cultivars) as moderately resistant, and 274 varieties (28 landraces, 246 cultivars) as susceptible. In the 2023/2024 growing season, the distribution shifted slightly, with 11 varieties (5 landraces, 6 cultivars) classified as highly resistant, 66 varieties (34 landraces, 32 cultivars) as moderately resistant, and 267 varieties (26 landraces, 241 cultivars) as susceptible. Notably, ‘Gaoyou 2018’ was consistently identified as an exceptional germplasm resource for PHS resistance breeding across both seasons (Table 1).

### 2.2. Identification of Molecular Markers for PHS Resistance

**Figure 1:**
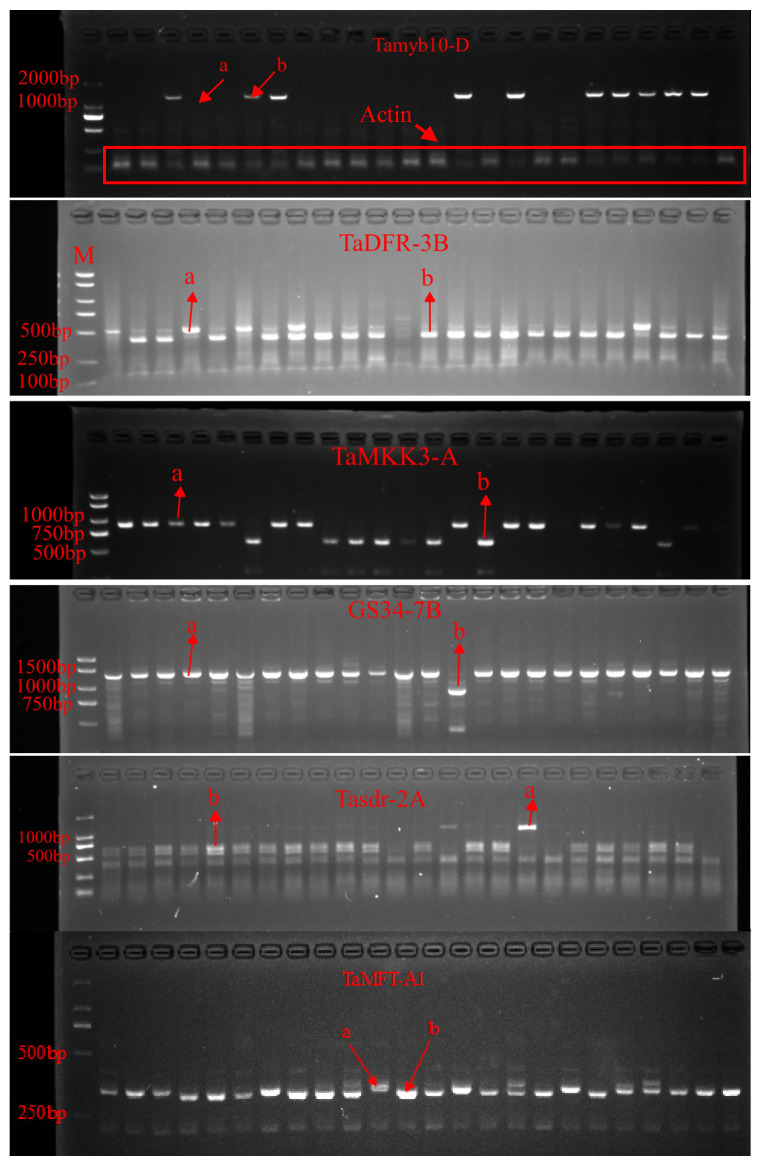
Agarose gel electrophoresis patterns of six molecular markers associated with PHS resistance in wheat. Lanes from left to right represent the following wheat varieties: Qiule 6, Shangmai 156, Zhongnong 867, Guanmai 2, Xinong 1125, Bainong 4199, Xinmai 58, Zhongmai 1818, Bainong 418, Anmai 29, Huayumai 1, Jinyongfeng 99, Jiyanmai 20, Nongda 753, Zhoumai 36, Fengdecunmai 23, Aimai 180, Fanyumai 18, Aimai 24, Dapingyuan 3, Qiule 168, Zhengmai 369, Fengdecunmai 21, and Dingyan 189. ‘a’ and ‘b’ represent different haplotypes for each molecular marker.

Molecular marker analysis was conducted on 344 white-grained wheat varieties to investigate PHS resistance-related genes. Figure 1 illustrates the agarose gel electrophoresis band patterns for six molecular markers associated with PHS resistance. Tamyb10-D1 [10,12], TaDFR-3B [11], TaMKK3-A [13], GS34-7B [16], Tasdr-2A [17], and TaMFT-A1 [18] are shown in Figure 1. Figure 1 illustrates the agarose gel electrophoresis band patterns for six molecular markers associated with PHS resistance. For Tamyb10-D1, the presence (1353 bp, haplotype a) or absence (haplotype b) of the fragment was observed. TaDFR-3B showed a 526 bp fragment for haplotype a and 102 bp + 432 bp fragments for haplotype b. TaMKK3-A displayed an 887 bp fragment for haplotype a and 605 bp + 282 bp fragments for haplotype b. GS34-7B exhibited a 1310 bp fragment for haplotype a and 900 bp + 410 bp fragments for haplotype b. Tasdr-2A showed an 1146 bp fragment for haplotype a and 528 bp + 618 bp fragments for haplotype b. For TaMFT-A1, a subtle size difference was observed between haplotype a (331 bp) and haplotype b (319 bp). Haplotype analysis revealed diverse distributions of a and b haplotypes among the varieties for each molecular marker. For Tamyb10-D1, 293 varieties exhibited the a haplotype, and 51 exhibited the b haplotype. TaDFR-3B showed 93 varieties with the a haplotype and 251 with the b haplotype. TaMKK3-A had 199 varieties with the a haplotype and 145 with the b haplotype. For GS34-7B, 291 varieties displayed the a haplotype, and 53 displayed the b haplotype. Tasdr-2A exhibited 28 varieties with the a haplotype and 316 with the b haplotype. Lastly, TaMFT-A1 showed a nearly equal distribution, with 164 varieties having the a haplotype and 180 the b haplotype. These results indicate substantial genetic diversity within the studied population with respect to PHS resistance-related genes, highlighting the complex nature of PHS resistance in wheat (Table S3).

### 2.3. Identification of Superior Haplotypes for PHS Resistance in Each Molecular Marker

The pre-harvest sprouting (PHS) rates for different haplotypes of the six molecular markers were evaluated separately for the 2022/2023 and 2023/2024 growing seasons. In the 2022/2023 season, Tamyb10-D1 haplotypes a and b showed PHS rates of 65.3% and 71.0%, respectively; TaDFR-3B exhibited rates of 61.1% (a) and 68.0% (b); TaMKK3-A displayed rates of 72.9% (a) and 56.9% (b); GS34-7B demonstrated rates of 70.5% (a) and 42.1% (b); Tasdr-2A showed rates of 42.0% (a) and 68.3% (b); and TaMFT-A1 exhibited rates of 73.6% (a) and 59.4% (b). In the 2023/2024 season, the rates were as follows: Tamyb10-D1, 62.5% (a) and 75.1% (b); TaDFR-3B, 54.0% (a) and 68.2% (b); TaMKK3-A, 70.1% (a) and 56.6% (b); GS34-7B, 68.5% (a) and 41.7% (b); Tasdr-2A, 46.2% (a) and 66.0% (b); and TaMFT-A1, 71.2% (a) and 58.2% (b). Based on these results, the findings for TaMKK3-A, GS34-7B, Tasdr-2A, and TaMFT-A1 corroborated previous research [13,16,17,18]. However, the results for Tamyb10-D1 and TaDFR-3B diverged from earlier studies [11,12]. This discrepancy may be attributed to the exclusive use of white-grained wheat varieties in the current study. Consequently, these two markers were excluded from subsequent statistical analyses.

Linear regression analysis showed that the haplotypes of Tamyb10-D, TaDFR-3B, TaMKK3-A, GS34-7B, Tasdr-2A, and TaMFT-3A explained 1.8%, 3.8%, 9.1%, 17.3%, 6.9%, and 8% of the phenotypic variation, respectively. These phenotypic explanation rates were based on the average spike germination rates over the 2022/2023 and 2023/2024 seasons.

**Figure 2:**
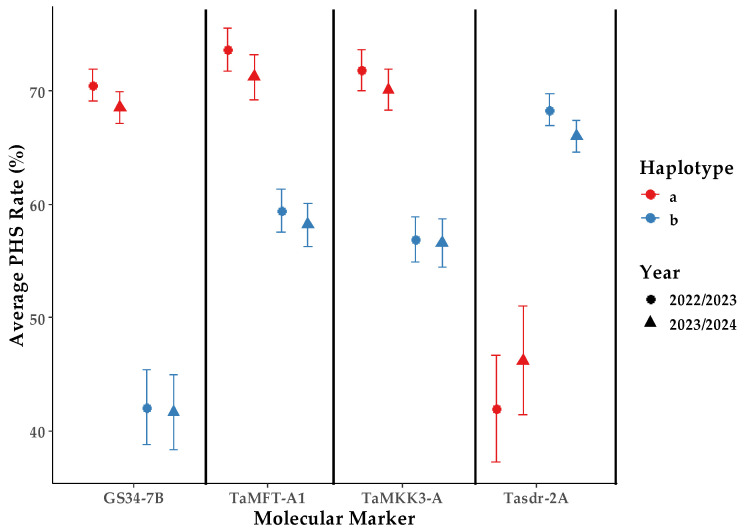
Average PHS rate by molecular marker, haplotype, and year. ‘a’ and ‘b’ represent different haplotypes for each molecular marker.

Mann–Whitney U tests were conducted using IBM SPSS Statistics 22 software to assess the significance of PHS rate differences between haplotypes of TaMKK3-A, GS34-7B, Tasdr-2A, and TaMFT-A1. The analysis revealed highly significant differences in two-growing-season average PHS rates between allelic haplotypes for all four markers (Figure 2, Table 2). Notably, the GS34-7B haplotype b exhibited the lowest two-growing-season average PHS rate, suggesting that the TaGASR34 gene plays a crucial role in reducing PHS susceptibility in white-grained wheat.

### 2.4. Significance Analysis of Different PHS Gene Haplotype Combinations and Variety of PHS Rates

Analysis of the test materials revealed 15 distinct genotypes resulting from various haplotype combinations of four molecular markers: TaMKK3-A, GS34-7B, Tasdr-2A, and TaMFT-A1. Notably, the TaMKK3-4Aa + GS34-7Bb + Tasdr-2Aa + TaMFT-A1b genotype was not observed in the population. The distribution frequency and average PHS rates for these genotypes are presented in Table 3. Type XV was the most prevalent, accounting for 29.1% of the population, while Type II was uniquely identified in the variety Bainong AK58.

A multiple comparison analysis was conducted on seven haplotype combinations, each represented by more than eight varieties (Table 3). Notably, the results demonstrated remarkable consistency between the 2022/2023 and 2023/2024 growing seasons. Type IX (TaMKK3-Ab + GS34-7Bb + Tasdr-2Aa + TaMFT-A1b) consistently exhibited the lowest average PHS rates over both growing seasons. Comparative analysis of genotypes differing only at the GS34-7B locus (X vs. XII and XI vs. XV) indicated that GS34-7Bb conferred significantly stronger PHS resistance than GS34-7Ba. Similarly, comparisons of genotypes varying only in TaMFT-A1 alleles (X vs. XI, XII vs. XIV, and XIII vs. XV) revealed that TaMFT-A1b had a significantly different effect on PHS resistance compared to TaMFT-A1a. Examination of IX and X for Tasdr-2A alleles showed no significant difference in PHS resistance between Tasdr-2Aa and Tasdr-2Ab. For TaMKK3-4A, a comparison of XII and XIII demonstrated that TaMKK3-Ab had a significantly different PHS resistance effect than TaMKK3-4Aa, while no significant difference was observed between XIV and XV. This discrepancy may be attributed to the presence of TaMFT-A1b in XII and XIII and TaMFT-A1a in XIV and XV. Consequently, combinations IX and X were identified as advantageous genotypes for PHS resistance in white-grained wheat.

Further investigation of the 10 varieties with two growing seasons average PHS rates below 20% (mentioned in Section 2.1) revealed that their genotypes were predominantly Types IX and X (Table 1). Specifically, four varieties belonged to Type IX and six to Type X, underscoring the importance of these genotypic combinations in conferring strong PHS resistance.

### 2.5. Correspondence Analysis Results of Different PHS Gene Haplotype Combinations and Variety of PHS Resistance Levels

**Figure 3:**
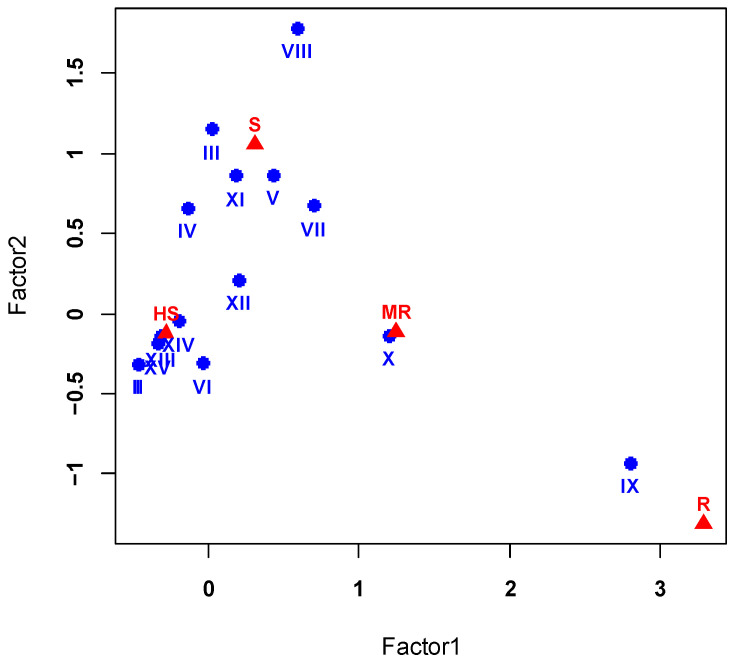
Correspondence analysis plot of PHS gene haplotype combinations and variety of PHS resistance levels. “●”: I–XV represent PHS gene haplotype combinations, corresponding to those in Table 3. “▲”: PHS resistance levels (R: resistant; MR: moderately resistant; S: susceptible; HS: highly susceptible).

A correspondence analysis was conducted based on the association probability matrix between 15 PHS gene haplotype combinations and five categories of a variety of PHS resistance levels. Non-zero eigenvalues ([min(r,c) − 1 = 3]) were extracted from the covariance matrix in accordance with correspondence analysis principles. As shown in Table 4, the first and second factors accounted for 66.8% and 30.0% of the total variance, respectively, with a cumulative contribution of 96.8%. Consequently, these two factors were selected for further analysis. Figure 3 illustrates the results of the correspondence analysis. The 15 haplotype combinations clustered into three main categories: resistant (R), moderately resistant (MR), and susceptible/highly susceptible (S and HS). PHS-resistant varieties (R) showed a strong association with the previously identified Type IX haplotype combination (TaMKK3-Ab + GS34-7Bb + Tasdr-2Aa + TaMFT-A1b). Moderately PHS-resistant varieties (MR) corresponded closely with Type X (TaMKK3-Ab + GS34-7Bb + Tasdr-2Ab + TaMFT-A1b). The remaining 13 haplotype combination types were predominantly associated with PHS-susceptible (S) and highly PHS-susceptible (HS) varieties.

These findings corroborate our earlier analysis, which identified advantageous genotypes for PHS resistance in white-grained wheat. The clear association between specific haplotype combinations and PHS resistance levels underscores the potential of these genetic markers for breeding PHS-resistant wheat varieties.

### 2.6. Temporal Distribution of Superior PHS Gene Haplotypes and Advantageous Combinations in Wheat Varieties

**Figure 4:**
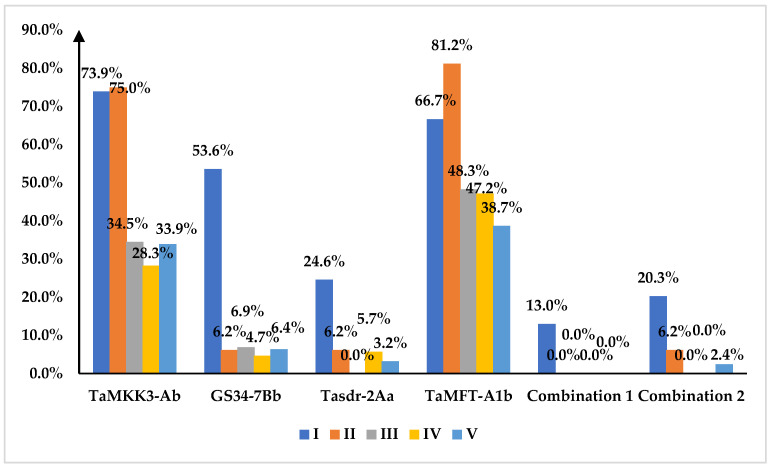
Proportion of superior haplotypes of molecular markers for PHS resistance and their advantageous combinations in wheat varieties from different eras. Combination 1: TaMKK3-Ab + GS34-7B + Tasdr-2Aa + TaMFT-A1b; combination 2: TaMKK3-4Ab + GS34-7Bb + Tasdr-2Ab + TaMFT-A1b.

The wheat varieties were categorized into five eras based on their breeding periods: Era I (pre–1950), Era II (1951–1970), Era III (1971–1990), Era IV (1991–2010), and Era V (2011–2023). The 344 test materials were distributed across these eras as follows: 69, 16, 29, 106, and 124 varieties in Eras I to V, respectively. The prevalence of superior haplotypes varied across eras. For TaMKK3-Ab, the proportions from Era I to V were 73.9%, 75%, 34.5%, 28.3%, and 33.9%, respectively. GS34-7Bb showed a marked decline after Era I, with proportions of 53.6%, 6.2%, 6.9%, 4.7%, and 6.4% across the five eras. Tasdr-2Aa exhibited a similar trend, accounting for 24.6%, 6.2%, 0.0%, 5.7%, and 3.2% from Era I to V. TaMFT-A1b maintained relatively higher frequencies, representing 66.7%, 81.2%, 48.3%, 47.2%, and 38.7% across the eras. Notably, the advantageous PHS-resistant superior haplotype combinations, namely combination 1 (TaMKK3-Ab + GS34-7B + Tasdr-2Aa + TaMFT-A1b) and combination 2 (TaMKK3-Ab + GS34-7Bb + Tasdr-2Ab + TaMFT-A1b), were predominantly found in Era I varieties (Figure 4).

Overall, the distribution frequencies of all four superior haplotypes showed declining trends over time, albeit to varying degrees. TaMKK3-Ab and TaMFT-A1b frequencies decreased significantly post-1970s, while Tasdr-2Aa and GS34-7Bb frequencies dropped sharply after the 1950s. The prevalence of superior haplotype combinations 1 and 2 also diminished in subsequent eras, with a particularly noticeable decline in Eras IV and V compared to Era I.

## 3. Discussion

Previous research by Mares and Mrva [19] has demonstrated that PHS resistance in wheat is associated with multiple gene loci distributed throughout the genome. Torada et al. [20] further corroborated this finding through QTL analysis, identifying key gene loci related to wheat PHS resistance and highlighting the potential value of molecular markers in PHS-resistant breeding. In the present study, we analyzed six PHS-related molecular markers and found that different haplotypes at four loci—TaMKK3-A, GS34-7B, Tasdr-2A, and TaMFT-A1—exhibited highly significant differences (*p* < 0.01) in PHS rates. A combined analysis of different allele combinations and their relationship with PHS revealed that GS34-7Bb and TaMFT-A1b had significant positive effects on PHS resistance. The positive effect of Tasdr-2Aa was not statistically significant, while the impact of TaMKK3-Ab requires further investigation. Notably, the b-type haplotype of GS34-7B consistently exhibited the lowest PHS rate in both the 2022/2023 and 2023/2024 growing seasons. This finding underscores the important role of the TaGASR34 gene in conferring PHS resistance in white-grained wheat [15] and aligns with previous reports of a significant association between the GS34-7B marker and PHS resistance [16]. Interestingly, the effects of superior haplotypes of Tamyb10-D1 and TaDFR-3B observed in this study diverged from previously reported results. This discrepancy may be attributed to our exclusive use of white-grained wheat materials, whereas previous studies included red-grained wheat varieties. Earlier research has established that Tamyb10 and TaDFR genes are involved in the synthesis of seed coat anthocyanins, and seed coat color is closely associated with PHS resistance [10,11,12,21]. Consequently, the effects of these two genes may manifest differently in white-grained wheat populations. These findings not only contribute to our understanding of the genetic basis of PHS resistance but also highlight the importance of considering grain color when interpreting the effects of PHS-related genes. Further research is needed to elucidate the specific mechanisms by which these genes influence PHS resistance in white-grained wheat.

PHS is a complex trait controlled by multiple genes and influenced by various allelic variations, underscoring the importance of joint analysis of superior allelic variations in wheat PHS-related genes. Zhang et al. [22] demonstrated through molecular marker analysis that in Chinese wheat mini-core collections and landraces, markers on the short arm of chromosome 3A (Xbarc57, Xbarc294, Xbarc310, Xbarc321) and a gene marker on 3BL (Vp1-b2) were significantly associated with seed dormancy traits. The combination of these five markers enhanced the accuracy of seed dormancy assessment, collectively explaining 95.9% of the genetic variation. In the present study, we conducted the first systematic analysis of the effects of different combinations of four gene haplotypes, TaMKK3-A, GS34-7B, Tasdr-2A, and TaMFT-A1, on PHS resistance. We identified 15 distinct haplotype combinations of PHS genes and performed significance analysis and correspondence analysis on these combinations using the average PHS rate and PHS resistance grade values of the test materials over two growing seasons. Both analyses revealed that the superior allelic variations of all four genes exhibited significant additive effects in enhancing PHS resistance. While the effect of a single gene’s superior allelic variation on reducing PHS rate is limited (e.g., the lowest average PHS rate of GS34-7B haplotype combinations over two growing seasons was 41.5%), pyramiding multiple superior allelic variations in the same combination can significantly reduce PHS rate. Notably, the combination types TaMKK3-Ab + GS34-7Bb + Tasdr-2Aa + TaMFT-A1b and TaMKK3-Ab + GS34-7Bb + Tasdr-2Ab + TaMFT-A1b demonstrated significant advantages in improving PHS resistance. The discovery of this multigene synergistic effect is crucial for understanding the complex genetic mechanism of PHS resistance and aligns with the multigene regulatory model of PHS resistance proposed by Tan et al. [23]. Our findings have direct application value for PHS-resistant breeding in white-grained wheat. The two identified advantageous gene combinations can serve as breeding targets, while the 10 superior performing varieties discovered, especially Xuzhou 438 and Gaoyou 2018, can be utilized as important germplasm resources. These results provide a solid theoretical foundation and practical guidance for future molecular breeding efforts aimed at improving PHS resistance in wheat. The identified gene combinations and superior varieties offer valuable tools for breeders to develop white-grained wheat cultivars with enhanced PHS resistance, potentially leading to significant improvements in wheat quality and yield stability under challenging environmental conditions.

## 4. Materials and Methods

### 4.1. Plant Materials

This study utilized a germplasm panel of 344 wheat varieties, comprising 65 landraces and 279 improved cultivars, which were provided by the Wheat Molecular Breeding Team at the Institute of Crop Molecular Breeding, Henan Academy of Agricultural Sciences. Field evaluations were conducted over two consecutive growing seasons (2022/2023 and 2023/2024) at the Henan Modern Agriculture Research and Development Base (113.707° E, 35.011° N). A randomized complete block design with two replications was employed. Each accession was manually sown in two-row plots (2.0 m length, 0.03 m intra-row spacing) to ensure precise seed placement. Agronomic management practices adhered to local recommendations for optimal wheat production in the region.

### 4.2. Pre-Harvest Sprouting Evaluation

The pre-harvest sprouting (PHS) evaluation method was adapted from the Agricultural Industry Standard of the People’s Republic of China NY/T 1939-2009 [24]. At the wax ripeness stage, 20 representative main stem spikes were selected from each replication, cut 20 cm below the spike neck, and stored at −20 °C until all materials were harvested. For the PHS treatment, germination was defined as visible rupture of the seed coat at the embryo. The total number of grains and the number of germinated grains per spike were counted. The spike sprouting (SS) rate was calculated as the percentage of germinated grains and expressed as the average of two replications. Concurrently, the control variety ‘Zhoumai 18’ was evaluated using the same method. The relative germination index (I) was calculated using the formula: I = X1/X2, where X1 is the SS rate of the test sample and X2 is the SS rate of the control sample. PHS resistance levels were determined based on the relative germination index (I) according to the criteria presented in Table S1.

### 4.3. Detection of Molecular Markers Associated with PHS Resistance

Genomic DNA was extracted from fresh leaf samples (0.2–0.3 g) of each variety using the method described by Gawel and Jarret [25]. Samples were pre-cooled with liquid nitrogen, ground, and transferred to 1.5 mL microcentrifuge tubes for DNA extraction. Table 5 summarizes the molecular marker information, including primer sequences and annealing temperatures. For Tamyb10-D1, the presence (haplotype a) or absence (haplotype b) of a 1353 bp fragment was detected. To ensure the quality of genomic DNA and PCR conditions, an internal reference gene (Actin) was amplified simultaneously using the following primers: forward 5′-GCTCGACTCTGGTGATGGTG-3′ and reverse 5′-AGCAAGGTCCAAACGAAGGA-3′, yielding a 150 bp product.

All primers were synthesized by Shanghai Sangon Biotech Co., Ltd., Shanghai, China PCR reactions were performed in a total volume of 20 μL, containing 80 ng template DNA, 0.5 μmol/L each of forward and reverse primers, 10 μL Fast Mighty Mix (Mona Biotech Co., Ltd., Suzhou, China ), and nuclease-free water to reach the final volume. PCR amplification programs and subsequent enzyme digestion of PCR products were carried out according to the protocols described in the respective reference literature for each marker.

For Tamyb10-D1 and Actin, a multiplex PCR approach was optimized to simultaneously amplify both targets. The resulting PCR products and digested fragments were separated by electrophoresis on 2% agarose gels for most markers. However, for TaMFT-A1, where the difference between haplotype a (331 bp) and b (319 bp) is minimal, we employed high-resolution electrophoresis using 3% agarose gels to ensure accurate distinction. Known controls for each haplotype were included on every gel. All gels were visualized under UV light and documented using a gel imaging system.

### 4.4. Data Analysis

Narrow-sense heritability (h^2^) is defined as the ratio of additive genetic variance (VA) to phenotypic variance (VP): h^2^ = V_A_/V_P_. Calculation steps: (a) Estimate phenotypic variance (VP): This is typically obtained through variance analysis of phenotypic values for all individuals in the population. (b) Estimate additive genetic variance (VA): This usually requires specific experimental designs, such as parent-offspring regression, sibling analysis, or twin studies. (c) Calculate the ratio: Divide the estimated VA by VP.

All statistical analyses were performed using IBM SPSS Statistics software (version 22). General linear model (GLM) analysis: We employed SPSS’s GLM procedure to analyze significant differences. The average spike germination rates from the 2022/2023 and 2023/2024 seasons were used as the dependent variable. Haplotypes of each gene (Tamyb10-D, TaDFR-3B, TaMKK3-A, GS34-7B, Tasdr-2A, and TaMFT-3A) were set as fixed factors. To avoid multicollinearity issues, each gene was analyzed separately. Type III sum of squares was used for computation, as it is appropriate for unbalanced designs. Levene’s test was conducted to assess the homogeneity of variance assumption. Bonferroni correction was applied for multiple comparisons to control the Type I error rate. F-values, degrees of freedom, and *p*-values were reported to indicate significance levels. The level of statistical significance was set at *p* < 0.05 for all analyses. Phenotypic variation calculation: The phenotypic variation explained by each gene’s haplotype was calculated using SPSS’s linear regression procedure. The average spike germination rate served as the dependent variable, while gene haplotypes were input as categorical independent variables using dummy coding. R^2^ values were reported for each model and converted to percentages to represent the phenotypic variation explained. Adjusted R^2^ values were also reported to account for the number of variables in the models.

Correspondence analysis: SPSS’s correspondence analysis procedure was used to explore associations between haplotypes and phenotypes. Input data consisted of haplotype frequencies and corresponding spike germination rate categories. Cumulative inertia was reported to evaluate the explanatory power of the analysis. Two-dimensional correspondence analysis plots were generated to visually represent the associations between haplotypes and phenotype categories.

## 5. Conclusions

This comprehensive study conducted a joint analysis of PHS resistance phenotype identification and PHS-resistant gene identification on 344 white-grained wheat varieties. Our findings reveal significant genetic variation in wheat PHS resistance among the test materials, providing valuable insights into the genetic architecture of this complex trait. We successfully elucidated the distribution patterns of four PHS resistance-related gene haplotypes (TaMKK3-A, GS34-7B, Tasdr-2A, and TaMFT-A1) in white-grained wheat. Through detailed analysis, we examined the effects of different allelic variations of these four genes and their various combination types on PHS resistance. Notably, our results demonstrate significant additive effects of superior haplotype combinations on PHS resistance. Two advantageous genotypes for PHS resistance in white-grained wheat were identified: TaMKK3-Ab + GS34-7Bb + Tasdr-2Aa + TaMFT-A1b and TaMKK3-Ab + GS34-7Bb + Tasdr-2Ab + TaMFT-A1b. These combinations show particular promise for enhancing PHS resistance in breeding programs. Interestingly, we observed a temporal decline in the distribution frequency of superior haplotypes of PHS genes and the two advantageous haplotype combinations, albeit to varying degrees. These findings have significant implications for PHS-resistant breeding strategies in white-grained wheat, particularly in the Huanghuai winter wheat region.

## Figures and Tables

**Table 1:** Five varieties with PHS rates < 20% in both 2022/2023 and 2023/2024 growing seasons and their genotypes.

Varieties	TaMKK3-4A	GS34-7B	Tasdr-2A	TaMFT-A1	2022/2023 PHS Rate (%)	2023/2024 PHS Rate (%)
QitouFuMai (Shanxian1)	b	b	a	b	11.5	17.7
Qisiniu	b	b	a	b	18.4	19.8
Baixiantiao	b	b	a	b	5.6	15.8
Baimai (Xiangcheng)	b	b	a	b	12.3	19.3
Ermangmai (Biyang1)	b	b	b	b	16.0	19.8
Gaoyou2018	b	b	b	b	18.5	4.4

‘a’ and ‘b’ represent different haplotypes for each molecular marker.

**Table 2:** Significance analysis of two-season average PHS rates between different haplotypes of PHS-related molecular markers in 2022/2023 and 2023/2024.

PHS Gene	Molecular Marker	Haplotypes	Number of Varieties	2022/2023 Average PHS Rate (%)	2023/2024 Average PHS Rate (%)
*TaMKK3-A*	TaMKK3-A	a	199	71.8 ± 1.8	70.1 ± 1.8
		b	145	56.9 ± 2.0 **	56.6 ± 2.1 **
*TaGASR34*	GS34-7B	a	291	70.5 ± 1.4	68.5 ± 1.4
		b	53	42.1 ± 3.3 **	41.7 ± 3.3 **
*Tasdr*	Tasdr-2A	a	28	42.0 ± 4.7 **	46.2 ± 4.8 **
		b	316	68.3 ± 1.4	66.0 ± 1.4
*TaMFT*	TaMFT-A1	a	164	73.6 ± 1.9	71.2 ± 2.0
		b	180	59.4 ± 1.9 **	58.2 ± 1.9 **

** means highly significant at the *p* < 0.001 level. ‘a’ and ‘b’ represent different haplotypes for each molecular marker.

**Table 3:** Average PHS rates and distribution frequencies of various PHS gene haplotype combinations during the 2022/2023 and 2023/2024 growing seasons.

Type	Haplotype Combinations	Number of Varieties	Distribution Frequency (%)	2022/2023 Average PHS Rate (%)	2023/2024 Average PHS Rate (%)
I	TaMKK3-Ab + GS34-7Bb + Tasdr-2Aa + TaMFT-A1a	2	0.6	72.2	77.7
II	TaMKK3-4Aa + GS34-7Bb + Tasdr-2Aa + TaMFT-A1a	1	0.3	79.7	97.1
III	TaMKK3-4Aa + GS34-7Ba + Tasdr-2Aa + TaMFT-A1a	2	0.6	50.5	55.7
IV	TaMKK3-4Aa + GS34-7Ba + Tasdr-2Aa + TaMFT-A1b	3	0.9	63.2	64.3
V	TaMKK3-Ab + GS34-7Ba + Tasdr-2Aa + TaMFT-A1a	5	1.5	45.1	55.6
VI	TaMKK3-4Aa + GS34-7Bb + Tasdr-2Ab + TaMFT-A1a	6	1.7	57.0	58.7
VII	TaMKK3-Ab + GS34-7Ba + Tasdr-2Aa + TaMFT-A1b	6	1.7	43.5	43.2
VIII	TaMKK3-4Aa + GS34-7Bb + Tasdr-2Ab + TaMFT-A1b	7	2.0	45.1	36.9
IX	TaMKK3-Ab + GS34-7Bb + Tasdr-2Aa + TaMFT-A1b	9	2.6	19.3 ± 7.3 A	22.3 ± 7.6 A
X	TaMKK3-Ab + GS34-7Bb + Tasdr-2Ab + TaMFT-A1b	18	5.2	35.2 ± 5.2 A	33.3 ± 5.3 A
XI	TaMKK3-Ab + GS34-7Bb + Tasdr-2Ab + TaMFT-A1a	10	2.9	54.0 ± 7.0 B	54.7 ± 7.2 B
XII	TaMKK3-Ab + GS34-7Ba + Tasdr-2Ab + TaMFT-A1b	51	14.8	58.0 ± 3.1 B	57.6 ± 3.2 B
XIII	TaMKK3-4Aa + GS34-7Ba + Tasdr-2Ab + TaMFT-A1b	86	25.1	71.6 ± 2.4 C	70.2 ± 2.4 C
XIV	TaMKK3-Ab + GS34-7Ba + Tasdr-2Ab + TaMFT-A1a	44	12.8	75.5 ± 3.3 CD	73.5 ± 3.4 CD
XV	TaMKK3-4Aa + GS34-7Ba + Tasdr-2Ab + TaMFT-A1a	94	27.3	77.8 ± 2.3 CD	73.4 ± 2.3 CD

Multiple comparison analysis was performed on 7 haplotype combinations with more than 8 varieties each. Different uppercase letters indicate significant differences (*p* < 0.05).

**Table 4:** Eigenvalues, contribution rates, and cumulative contribution rates of each Factor.

Factor	Eigenvalues	Contribution (%)	Cumulative Contribution (%)
1	0.37	66.8	66.8
2	0.17	30.0	96.8
3	0.02	3.2	100

**Table 5:** Molecular marker information for wheat PHS genes.

Gene	Locus	Primer Sequence (5′-3′)	Allele	Fragment Size	Annealing Temperature (°C)	References
*Tamyb10*	Tamyb10-D1	F: TAGGCCAACACCTTCTAAACG	Tamyb10-D1a	—	60	[10,12]
		R: AGGCACACCAGCTTATTTGG	Tamyb10-D1b	1353 bp		
*TaDFR*	TaDFR-3B	F: TGCGGTCTGGCGGGGTACGT	TaDFR-Ba	526 bp	60	[11]
		R: ACGTCGAGAGAGAGAGGGAGGGG	TaDFR-Bb	102 bp 432 bp		
*TaMKK3-A*	TaMKK3-A	F: CACCAAAGAATAGAAATGCTCTCT	TaMKK3-Aa	887 bp	62	[13]
		R: AGGAGTAGTTCTCATTGCGG	TaMKK3-Ab	605 bp 282 bp		
*TaGASR34*	GS34-7B	F: ACACCTCGGTTCAATGCC	GS34-7Ba	1310 bp	60	[16]
		R: CCTTCTTGTACTGCGTCCC	GS34-7Bb	900 bp 410 bp		
*Tasdr*	Tasdr-2A	F: CGTCGGCAGACATCGACTCC	TaSdr-A1a	1146 bp	59	[17]
		R: GAAGCTCACTAGCTCAGAACACGC	TaSdr-A1b	528 bp 618 bp		
*TaMFT*	TaMFT-A1	F: GAGCAAACATGTCCCGGTTCGTT	TaMFT-A1a	331 bp	57	[18]
		R: ATCACCATGCACACACATACATAAATCACC	TaMFT-Alb	319 bp		

## Data Availability

Data is contained within the article and Supplementary Materials.

## Supplementary Materials

The following supporting information can be downloaded at https://www.mdpi.com/article/10.3390/ijms26020728/s1.
